# Naming Global Health

**DOI:** 10.4269/ajtmh.2011.10-0602

**Published:** 2011-01-05

**Authors:** David Brett-Major

**Affiliations:** US Military Tropical Medicine; Navy Medicine Manpower Personnel Training and Education Command and; Division of Tropical Public Health; Department of Preventive Medicine and Biometrics; Uniformed Services University; 8901 Wisconsin Avenue; Bethesda, MD 20889; E-mail: dmbrettmajor@gmail.com

Dear Sir:

During my senior year in college, a professor accused our small class of a topology reading seminar of trying to lord power through language. He argued, on several interminable occasions, that, instead of choosing words that we knew to be terms of art, we should speak as simply as possible. We should try to have any person understand and credit their thinking without punishing them for any failure of their vocabulary. Simplicity is beautiful. Language is beautiful whether simple or complex. I always have had trouble with that balance. However, making language complex has ancient roots. Knowing names that others do not secures possession. To name is to own. In the Judeo-Christian tradition, when man was granted dominion over creation, “and whatever the man called every living creature, that was its name” (Genesis 2:15).^1^ We do it with our children. My children have my name. We are doing this with global health. Wait, we are doing it with international health. That is, we are doing it with humanitarian assistance. At least we are not doing it with health aid, health development, relief, tropical public health, or developing world health. Actually, I think that last one is partially my fault. However, maybe not; maybe I saw it in a brochure by someone else and stole it.

I have been doing that quite a bit lately, stealing names. Someone near me starts using new terms for areas in which I already am involved. Therefore, I take the name and help it find its way into the text of my brochure. It is a gentler practice than keeping that someone from growing whatever he is trying to grow over there, even if using the same water supply. No dams. No diverting irrigation channels. No poaching upstream land. Just name stealing. There is sufficient poverty and disease for everyone to work in decreasing them. I see the utility in naming to focus effort, enhance nuance, distinguish, and yet, remain eligible for multiple funding streams. However, being able to identify the roots of words is helpful as well. Work in the tropics and from the tropics spawned many of these disciplines. Additionally, the most egregious health disparities remain in the tropics. However, tropics and tropical are falling away from the language of what we do. We should be careful that our history, purpose, and so-named society fair better than them.
